# Extending the Continual Reassessment Method to accommodate step‐up dosing in Phase I trials

**DOI:** 10.1002/sim.9487

**Published:** 2022-06-05

**Authors:** Thomas M. Braun, Francois Mercier

**Affiliations:** ^1^ Department of Biostatistics University of Michigan Ann Arbor Michigan USA; ^2^ Department of Biostatistics Roche Innovation Centre Basel Switzerland

**Keywords:** adaptive clinical trial, Bayesian inference, conditional probability, dose escalation, fractionated dosing, up‐titration

## Abstract

The Continual Reassessment Method (CRM) was developed for Phase I trials to identify a maximum‐tolerated dose of an agent using a design in which each participant is treated with a single administration of the agent. We propose an extension of the CRM in which participants receive multiple administrations of an agent using a so‐called step‐up dosing procedure in which participants receive one or more administrations of lower doses of the agent before they receive their penultimate dose. We use methods developed for the CRM to model the probability of DLT for each administration, which leads to the use of conditional probability models to model the joint probability of DLT across multiple administrations. We compare our approach to two existing methods that use time‐to‐event modeling methods for modeling the probability of DLT. We demonstrate through simulations that our approach has operating characteristics similar to existing methods, but due to its foundations in the CRM, ours is simpler to implement than existing approaches and is therefore more likely to be adopted in practice.

## 1 INTRODUCTION

Phase I trials in oncology are designed to identify safe, and possibly effective, doses of prospective treatments for cancer. A number of Bayesian adaptive designs for Phase I trials have been developed over the past three decades, the first of which was the Continual Reassessment Method (CRM).^1^ In these designs, study participants are followed during a fixed period of time for the occurrence of a dose‐limiting toxicity (DLT), which is often defined as any treatment‐emergent adverse event of toxicity grade 3 or higher according to Common Terminology Criteria for Adverse Events (CTCAE) criteria.

In immunotherapy, DLTs often include cytokine release syndrome (CRS), which is a systemic inflammatory disease characterized by a massive release of cytokine.^2^ CRS can present with a variety of symptoms ranging from those that are mild to those that are life threatening, and sometimes fatal.^3^ Mild symptoms of CRS include fever, fatigue, nausea, vomiting, headache, rash, arthralgia, myalgia, and malaise. Several case reports have documented CRS in cancer patients treated with immune‐checkpoint inhibitors.^4^, ^5^, ^6^, ^7^, ^8^, ^9^, ^10^, ^11^

Recently, in the context of CAR T‐cells, Stein et al^12^ have proposed a model‐based approach to retrospectively characterize the kinetics of tisagenlecleucel, and its relationship with the emergence of CRS. In their work, they share concerns that co‐medication with steroids or tocilizumab may not suffice to mitigate CRS. Instead, in a more recent and physiological‐based model, Jiang et al^13^ suggest that a stepwise dosing of blinatumomab in patients with non‐Hodgkin's lymphoma may successfully alleviate release of interleukin‐6 and prevent CRS adverse events to occur.

To this end, a post‐marketing authorization study, listed on ClinicalTrials.gov with identifier NCT01029366, was designed to assess the benefit of fractionated dosing in adult patients with relapsed/refractory acute lymphocytic leukemia treated by CAR T‐cell therapy.^14^ Patients received either a low dose of tisagenlecleucel as a single infusion (LDS), or a high dose of tisagenlecleucel as a single infusion (HDS), or the high dose was split into fractions (HDF), with 10% of the dose delivered on day 1, 30% on day 2, and the remaining 60% on day 3. In the cohorts treated with a fractionated dosing approach, D2 and/or D3 doses were held if the patient experienced early signs of CRS, including fever. The frequency of grades 4 and 5 CRS was markedly lower in the HDF cohort (1 out of 20 patients) compared to the LDS (2 out of 9) or the HDS (3 out of 6) cohorts. As a result, fractionated or step‐up dosing (SUD) has been proposed in other programs^15^ and seems to be a promising treatment option to mitigate CRS. However, challenges arise in the design and analysis of DLT data collected during dose escalation studies involving fractionated dosing.

As the goal is now to identify a maximum tolerated dose (MTD) that is preceded by one or more lower doses, which we call a maximum‐tolerated schedule (MTS), the first challenge is to deal with probability of a DLT occurring after any of several consecutive dosing events. Both Colin et al^16^ and Fernandez et al^17^ proposed a logistic regression model with a Markov component for deal with DLTs observed over multiple cycles. Assuming that the drug was administered in consecutive cycles of similar length, an additive time homogeneous transition model could be specified as $\log(1-p_{ik})=-\alpha [d_{i1}-\rho d_{i,(k-1)}]-\beta d_{ik}$ where, for patient i receiving cycle k, $p_{ik}$ is the probability of toxicity, $d_{ik}$ is the scaled dose, and $d_{ik}=\sum_{k}d_{ik}$ is the cumulative administered scaled doses received. Respectively, the parameters α, β, and ρ capture the probability of a DLT on cycle 1, the effect of cumulative dose from previous cycles, and the dependency in short‐term toxicity outcomes between cycles (Markov term). A limitation of this model is that there must be sufficient cycles in the DLT assessment period to allow a plausible estimation of ρ and β. Also, Fernandez et al did not consider the setting in which dose varies across dosing events during the DLT assessment period, making their design unsuitable to the situation of fractionated dosing.

A second challenge in the design of fractionated dosing studies occurs because later planned administrations are omitted once a participant experiences a DLT after earlier administrations. Indeed, it is routine practice to discontinue or hold treatment in patients who experience DLT. If the intention is to develop a dose‐toxicity model akin to those used in the CRM, the missing administrations and resulting DLT outcomes will inflate the uncertainty associated with the model parameters and make the dose recommended for the next cohort less robust. In addition, by using SUD, we assume the dose of the first administration is lower than or equal to the dose of the second administration, which is lower than or equal to the dose of the third administration. Through this treatment plan, the absence of DLT associated with the second and/or third doses will lead to greater emphasis on DLT outcomes observed at lower doses, resulting in over‐conservative dose escalation and loss of the convergence benefits of the CRM.^18^

To circumvent this problem, Braun and colleagues^19^, ^20^ modeled DLT as an absorbing state that precludes any further administrations, using time‐to‐event concepts rather than logistic regression methods. As originally conceived, this work assumed that the hazard of DLT for a single administration increases linearly to a known future point in time and then decreases linearly to zero to a later point in time. The total hazard for several administrations is simply the sum of the hazards of the individual administrations that have been received, and the probability of DLT is based upon the survivor function derived from the cumulative hazard. By modeling the hazard, this approach moves away from a binomial likelihood and instead uses the likelihood of survival models, which accounts for partial follow‐up of participants, much like the time‐to‐event Continual Reassessment Method (TITE‐CRM)^21^ was developed to account for partial follow‐up in Phase I trials designed with the CRM.

In a similar vein, Gunhan et al^22^ recently modeled the probability of DLT through a hazard function for each administration like the methods of Braun and colleagues. However, Gunhan et al chose to relate the DLT hazard to a parametric pharmacokinetic (PK) model quantifying the cumulative drug exposure over time. Gerard and colleagues^23^ also created a design that integrates PK and pharmacodynamic (PD) models into the design. Given that in a more general setting, it may be unclear how to order consecutive administrations of doses with respect to their cumulative probabilities of DLT, Wages et al^24^ presented an approach to incorporate partial ordering constraints and used existing CRM modeling for each administration.

Like many adaptive Phase I trial methods, many view the statistical underpinnings of dose and schedule‐finding methods as too complex, a view which tends to hinder their implementation in practice. Thus, as an alternative to the time‐to‐event approaches cited earlier, we have adopted the longitudinal binary outcome view used by Fernandez et al. However, our model is based on the CRM, which is a widely accepted adaptive design for Phase I trials of single administrations. By using the CRM as a framework, we hope to reach our primary goal with a simpler, more accepted model, that performs as well as more complex methods, but is more likely to be adopted by a wider audience of clinical trialists. The underlying specifics of our design can be found in Section 2. Via simulation, we compare the operating characteristics of our design to the designs of Braun et al and Gunhan et al in Section 3, and we present our concluding thoughts in Section 4.

## 2 METHODS

### 2.1 Notation

We have a study designed to examine a set of J predefined treatment schedules, each of which is a series of K administrations of an investigational agent. The study will enroll a total of N participants, each of whom will be assigned to one of the J treatment schedules. We denote schedule j=1,2,…,J as ${𝒮}_{j}=\{D_{j1},D_{j2},\ldots ,D_{jK}\}$, in which $D_{jk}$ is the value for the dose of the agent given at administration k=1,2,…K in schedule j. We will describe how to select a value for $D_{jk}$ in a later section.

Our primary goal is to identify which treatment schedule has a probability of DLT at the end of follow‐up for the entire schedule closest to a targeted probability $p^{*}$. For k<K, we let $\tau_{k}$ denote the span of time between administrations k and k−1. For each administration, we let $0\leq t_{ik}\leq \tau_{k}$ denote the amount of follow‐up observed for participant i=1,2,…,N after receiving administration k, and we let $w_{ik}=t_{ik}/\tau_{k}$ denote the proportion of completed follow‐up.

At the start of administration k, we set the DLT indicator $Y_{ik}=0$, which changes to $Y_{ik}=1$ if participant i experiences a DLT before $\tau_{k}$. Once $Y_{ik}=1$, we also set $w_{ik}=1$, that is, we assume complete follow‐up occurs, reflecting a recommendation of treatment discontinuation after a patient experiences a DLT. Furthermore, once $Y_{ik}=1$, participant i receives no further planned administrations, while if $Y_{ik}=0$ when $t_{ik}=\tau_{k}$, participant i receives their next administration.

### 2.2 Model

For each administration, we model the marginal probability of DLT with a model traditionally used in the CRM, known as the “power” or “empiric” model. First, assuming that patient i is assigned to schedule j, we let $d_{ik}=D_{jk}\in (0,1)$ be the value assigned to the dose given to participant i at administration k, and we assume

(1) $$\begin{array}{ll} \pi_{i1}=Pr(Y_{i1}=1\;|\;d_{i1}) & =d_{i1}^{exp(\beta )} \end{array}$$

(2) $$\begin{array}{ll} \pi_{i2}=Pr(Y_{i2}=1\;|\;d_{i2}) & =d_{i2}^{exp(\beta -\theta_{2})}\\ \pi_{i3}=Pr(Y_{i3}=1\;|\;d_{i3}) & =d_{i3}^{exp(\beta -\theta_{2}-\theta_{3})}\\ \cdots & \\ \pi_{iK}=Pr(Y_{iK}=1\;|\;d_{iK}) & =d_{iK}^{exp(\beta -\overset{K}{\underset{k=2}{\sum }}\theta_{k})}, \end{array}$$

in which −∞≤β≤∞, $\theta_{k}\geq 0$ for k=2,3,…K. Note that we place no restriction on the value of β because the DLT probability for the first administration can take any value in [0,1]. However, we do place a non‐negativity constraint on each of $\theta_{2},\theta_{3},\ldots \theta_{K}$ to enforce an ordering constraint. Specifically, we assume that the cumulative probability of DLT cannot decrease with additional administrations. This restriction could be removed for another setting should this assumption not hold. However, less restriction on the parameters may also impact the ability to sufficiently identify those parameters, requiring continued focus on both appropriate prior distributions and the number of study participants needed to collect sufficient data to estimate those parameters.

Although our model can theoretically accommodate any number of administrations, most practical settings will examine a handful of administrations at most. Furthermore, we highlight that each administration k≥2 corresponds to an additional model parameter $\theta_{k}$. Given the relatively small sample sizes used in dose‐finding trials, the number of administrations will have to be limited without strong constraints or assumptions placed on model parameters. As a result, the remainder of this manuscript will focus upon our motivating example that studied K=3 administrations.

We will assume β has a normal prior distribution with mean $\mu_{1}$ and SD σ, while $\theta_{2}$ and $\theta_{3}$ each have exponential prior distributions with respective means $\mu_{2}$ and $\mu_{3}$. Note that β, $\theta_{2}$, and $\theta_{3}$ are *a priori* independent of each other, and we will present a systematic approach for selecting values for the four hyperparameters in Section 2.5.

Because further planned administrations are not given to participants who experience DLT, the observed data cannot be used to directly estimate $\pi_{i2}$ and $\pi_{i3}$. Instead, the observed data allow us to estimate the conditional probabilities of DLT for the second and third administrations, given no DLT was observed in all prior administrations. Nonetheless, the conditional probabilities are easy to generate from our model. Specifically, as derived in the Appendix, we have:

$$\begin{array}{ll} \phi_{i2} & =Pr(Y_{i2}=1\;|\;Y_{i1}=0,d_{i1},d_{i2})=\frac{\pi_{i2}-\pi_{i1}}{1-\pi_{i1}}.\;\\ \phi_{i3} & =Pr(Y_{i3}=1\;|\;Y_{i1}=Y_{i2}=0,d_{i1},d_{i2},d_{i3})=\frac{\pi_{i3}-\pi_{i2}}{1-\pi_{i2}}.\; \end{array}$$

### 2.3 Likelihood

Thus, at any point in the trial, we have enrolled a total of M≤N participants, who belong to one of three groups: those who have received the first administration, those who have received the first and second administrations, and those who have received all three administrations. We denote these three groups as ${𝒢}_{1},{𝒢}_{2}$, and ${𝒢}_{3}$, respectively. We let ${𝒟}_{1}$ denote the collective set of doses, DLT outcomes, and lengths of follow‐up for all participants in ${𝒢}_{1}$, with corresponding definitions for ${𝒟}_{2}$ and ${𝒟}_{3}$. Thus, each of the three groups has a respective likelihood equal to:

$$\begin{array}{ll} L_{1}(\beta \;|\;{𝒟}_{1}) & =\overset{M}{\underset{i=1}{\prod }}{\left[\pi_{i1}^{Y_{i1}}{\left(1-w_{i1}\pi_{i1}\right)}^{\left(1-Y_{i1}\right)}\right]}^{I(i\in {𝒢}_{1})}\;\\ L_{2}(\beta ,\theta_{2}\;|\;{𝒟}_{2}) & =\overset{M}{\underset{i=1}{\prod }}{\left[(1-\pi_{i1})\phi_{i2}^{Y_{i2}}{\left(1-w_{i1}\phi_{i2}\right)}^{\left(1-Y_{i2}\right)}\right]}^{I(i\in {𝒢}_{2})}\;\\ L_{3}(\beta ,\theta_{2},\theta_{3}\;|\;{𝒟}_{3}) & =\overset{M}{\underset{i=1}{\prod }}{\left[(1-\pi_{i2})\phi_{i3}^{Y_{i3}}{\left(1-w_{i1}\phi_{i3}\right)}^{\left(1-Y_{i3}\right)}\right]}^{I(i\in {𝒢}_{3})},\; \end{array}$$

which leads to the overall likelihood

(3) $$L(\beta ,\theta_{2},\theta_{3}\;|\;{𝒟}_{1},{𝒟}_{2},{𝒟}_{3})=L_{1}(\beta \;|\;{𝒟}_{1})\times L_{2}(\beta ,\theta_{2}\;|\;{𝒟}_{2})\times L_{3}(\beta ,\theta_{2},\theta_{3}\;|\;{𝒟}_{3}).$$

Note that the weights used in the likelihood are akin to those used in the time‐to‐event CRM (TITE‐CRM),^21^ which assume that DLTs occur uniformly during the follow‐up period, and are related to a cure model for the distribution of times to DLT.^25^

### 2.4 Specifying skeleton

Prior to study start, thought must be given to the numeric value assigned to each dose given at each administration. Given the complexity of selecting appropriate values for all of the JK=18 administrations, we have developed a systematic approach to identifying values that allow for generally good operating characteristics across many settings, as we will show in Section 3.

We start by assigning $D_{11}=\delta$, where δ is a value relatively close to zero and can be seen as an approximate probability of DLT for the lowest dose examined in the study. In our application, we use δ=0.03; see Table 1. For the remaining first doses of schedules 2,3,…J, we choose values that increase by an amount defined by an odds ratio $OR_{b}$ (“b” is for between‐schedules), that is, for j=2,3,…J,

$$\frac{D_{j,1}}{1-D_{j,1}}=OR_{b}\frac{D_{j-1,1}}{1-D_{j-1,1}}.$$

We then select two additional odds ratios $OR_{w1}$ and $OR_{w2}$ (“w” is for within‐schedules) such that for j=1,2,…J,

$$\frac{D_{j2}}{1-D_{j2}}=OR_{w1}\frac{D_{j1}}{1-D_{j1}},$$

and

$$\frac{D_{j3}}{1-D_{j3}}=OR_{w2}\frac{D_{j2}}{1-D_{j2}}.$$

Values of the odds ratios should be sufficiently large enough so that successive schedules have DLT probabilities that are distinct enough from each other and promote discrimination between them with the traditional sample sizes used in dose‐finding studies. We have found that appropriate values for each of the odds ratios are generally between 1.5 and 2.5, but they need to be determined in conjunction with the hyperparameter values described next. Selection of appropriate values for the odds ratios also can be informed by existing clinical information or previous dose‐escalation studies that might inform how doses vary in their DLT probabilities and the cumulative effects of repeated dosing. Nonetheless, like any regression model, estimation of model parameters is facilitated by variability in the predictor variable, which, in our setting, are the values assigned to each administration. Even if one believes, for example, that $OR_{w1}$ should be 1.1, such a study will be hard to implement without an unrealistically large number of participants, and doses with greater variability should be considered.

**TABLE 1:** Actual dose values, assigned skeleton values, and hypothetical true toxicity probabilities for motivating example presented in Section 3

	Administration			Administration					
	1	2	3	1	2	3			
Schedule	Actual dose values			Skeleton dose values					
1	0.006	0.018	0.018	0.03	0.05	0.05			
2	0.030	0.090	0.090	0.06	0.10	0.10			
3	0.090	0.270	0.270	0.10	0.16	0.16			
4	0.270	0.800	0.800	0.16	0.25	0.25			
5	0.800	2.400	2.400	0.25	0.36	0.36			
6	2.400	7.200	7.200	0.36	0.50	0.50			

	True DLT probabilities								
	Scenario 1			Scenario 2			Scenario 3		
1	**0.20**	**0.21**	**0.23**	0.10	0.11	0.13	0.02	0.03	0.05
2	0.25	0.29	0.34	**0.15**	**0.19**	**0.24**	0.07	0.10	0.14
3	0.31	0.37	0.45	0.21	0.27	0.35	**0.12**	**0.17**	**0.24**
4	0.37	0.45	0.56	0.27	0.35	0.46	0.17	0.24	0.34
5	0.45	0.57	0.73	0.35	0.47	0.63	0.24	0.34	0.48
6	0.56	0.73	0.95	0.46	0.63	0.85	0.34	0.48	0.67

	Scenario 4			Scenario 5			Scenario 6		
1	0.01	0.02	0.03	0.01	0.02	0.03	0.01	0.01	0.02
2	0.03	0.06	0.10	0.04	0.06	0.08	0.03	0.04	0.06
3	0.06	0.10	0.15	0.06	0.10	0.12	0.05	0.07	0.10
4	**0.08**	**0.15**	**0.24**	0.09	0.14	0.15	0.07	0.09	0.13
5	0.11	0.21	0.34	**0.13**	**0.20**	**0.26**	0.10	0.13	0.18
6	0.16	0.30	0.48	0.18	0.28	0.36	**0.14**	**0.19**	**0.27**

	Scenario 7								
1	0.25	0.35	0.45						
2	0.30	0.40	0.50						
3	0.36	0.46	0.56						
4	0.42	0.52	0.62						
5	0.50	0.60	0.70						
6	0.61	0.71	0.81						

*Note*: Boldface represents the optimal schedule, i.e. the schedule with toxicity probability closest to 0.25 after the third administration.

### 2.5 Specifying hyperparameters

In order to determine appropriate prior means for each of β, $\theta_{2}$, and $\theta_{3}$, we take Equations (1) and (2) and define $\eta_{jk}=Pr(Y_{i1}=1\;|\;D_{ik}=d_{jk})$, which is the same value for every participant. With regard to the prior mean for β, we identify an appropriate value through the values $D_{11},D_{21},\ldots ,D_{61}$ assigned to the set of first doses among the schedules. Based upon a second‐order Taylor series expansion for $\eta_{j1}=d_{j1}^{exp(\beta )}$, we outline in the Appendix that a suitable value for the prior mean is $\mu_{1}=\sum_{j=1}^{J}\log\left[-\log(D_{j1})\right]/J$, which is the average of the transformed dose values. We then select $\sigma =\sqrt{\mu_{1}}$, so that the exponent exp(β) in $\eta_{j1}$ has mean $exp(\mu_{1}+0.5\sigma^{2})=exp(1.5\mu_{1}).$

For the prior means of $\theta_{2}$ and $\theta_{3}$, we first select a value k≥1, such that $\eta_{j2}=k\eta_{j1}$ and $\eta_{j3}=k\eta_{j2}$, so that the probability of DLT within‐schedule is assumed to increase proportionally with each administration. First focusing on the prior mean for $\theta_{3}$, we start with the fact that $\eta_{j3}=\eta_{j2}^{exp(-\theta_{3})}$, which leads to $\theta_{3}=-\log[\log(\eta_{j3})/\log(\eta_{j2})]$. If we set $\eta_{j3}=p^{*}$ and given that $\eta_{j2}=\eta_{j3}/k$, we use the resulting equation for the prior mean of $\theta_{3}$, that is,

$$\mu_{\theta_{3}}=\frac{\log(p^{*})}{\log(p^{*})-\log(k)}.$$

By similar algebra, we find $\theta_{2}=-\log[\log(\eta_{j2})/\log(\eta_{j1})]$, which leads to the value

$$\mu_{\theta_{2}}=\frac{\log(p^{*})-\log(k)}{\log(p^{*})-2\log(k)}.$$

Using these four hyperparameter values, we generate many draws of β, $\theta_{2}$, and $\theta_{3}$ from their respective prior distributions, which allows us to compute many realizations of the probability of DLT for each administration of each schedule. Averaging over the realizations gives us an expected *a priori* probability of DLT for each administration of each schedule. These averages then allow us to see which schedule is now assumed to be the *a priori* best schedule and whether or not the prior distributions should be modified to produce suitable *a priori* DLT probabilities. For example, we would not want prior distributions that suggest that the first schedule is the best schedule, as this would likely be too informative and lead to poor operating characteristics if the true best schedule were the last schedule. To help the reader apply these ideas in practice, we will explore these thoughts in greater detail in Section 3 when presenting the prior distributions developed for the simulation study.

### 2.6 Dose assignment algorithm

Once all necessary study design parameters, including sample size, skeleton dose values, and prior distributions, have been identified, participant assignments are made adaptively as follows:

1. The first participant is assigned to the schedule with the lowest starting dose;
2. Once a new participant ℓ=2,3,…N, can be enrolled, the accrued data for participants i=1,2,…(ℓ−1) are used to compute the posterior distributions of probability of DLT at the end of follow‐up for each schedule;
3. Per a predefined stopping rule, if the accrued data suggest that the cumulative DLT probability after the follow‐up for the last administration in first schedule is unacceptably high, the study ends early and no further accrual occurs because all schedules are considered unsafe.
4. If the stopping rule is not met, the new participant is assigned to the schedule with cumulative posterior mean probability at the end of follow‐up closest to a desired target probability $p^{*}$, subject to any restrictions on assignments.
5. Repeat steps 2‐4 as each new participant is accrued, or until the study is stopped.
6. If the study has not stopped accrual, once participant N has completed their follow‐up:
  (a) Use all of the accrued data to compute the cumulative posterior mean probability of DLT at the end of follow‐up for each schedule;
  (b) Select the schedule with posterior mean probability closest to $p^{*}$ as the maximum tolerated schedule.

As suggested in step 3 above, Phase I trials often include a stopping rule when excessive toxicity is observed for the first schedule. Although stopping rules can be based upon posterior DLT probabilities, we have chosen instead to use a frequentist‐based stopping rule that is independent of the model used for computing DLT probabilities. Specifically, we use the observed number of DLTs seen for participants assigned to the first schedule to compute a one‐sided 95% confidence interval for the true DLT probability of the lowest schedule. If the lower bound of this confidence interval is higher than the targeted DLT probability $p^{*}$, the trial is stopped and future accrual is terminated. For example, the study would be stopped if four out of five participants assigned to schedule 1 experienced a DLT. This stopping rule is implemented in step 3 of the algorithm above and will be assessed in Section 3 with a setting in which all schedules have excessive probability of DLT.

In step 4 of the algorithm above, many possible restrictions on assignments can occur. For example, it is common to require that a schedule cannot be assigned to a participant until all lower schedules have been assigned to at least $N_{1}$ participants, or that participants must be enrolled in cohorts of size C, that is, all members of the same cohort must be given the same assignment. A common choice is C=3, which is adopted from the so‐called 3+3 algorithm.^26^ We could also require that at least $N_{2}$ participants must have completed their follow‐up on the same schedule before higher schedules can be assigned. Note that our parametric model, through the use of prior distributions, is able to estimate the DLT probabilities of later administrations without the direct observation of individuals who have received those administrations. Discomfort with decision‐making on incomplete data is mitigated directly through these restrictions.

Furthermore, one might also consider the entire posterior distribution, beyond the posterior mean, when making assignment decisions. This approach, commonly referred to as Escalation with Overdose Control (EWOC),^27^ examines how much mass of the posterior distribution lies above a threshold for each schedule's cumulative DLT probability. An upper bound is placed upon how much mass is acceptable and only schedules meeting this criterion are considered for assignment. Thus, it is possible that a schedule may have a posterior mean DLT probability that is closest to the targeted DLT probability, but its entire posterior distribution may be skewed or have too large a variance to be certain that the schedule is safe enough to assign to the next participant.

## 3 SIMULATIONS

### 3.1 Motivating example

Our motivating example is a study of six schedules (J=6) that follow a SUD plan. Each schedule consists of three administrations (K=3) of an experimental agent, each spaced apart by seven days ($\tau_{1}=\tau_{2}=7$), and it is assumed that the dose used in each administration is no more than the dose used in the preceding administration. The actual clinical doses under study are shown in the first six rows of Table 1. Participants are followed for an additional seven days after the third administration, for a total planned follow‐up of 21 days given to each participant. After each administration, patients are continually followed for the occurrence of a DLT, defined as any grade 3 or higher adverse event (per National Cancer Institute CTCAE v5.0), or occurrence of CRS grade 3 or above, according to the ASTCT consensus.^28^ If a participant experiences a DLT, all further planned administrations are withheld for that participant, and they are considered to have complete follow‐up.

### 3.2 Simulation details

Our study wishes to identify which schedule is associated with a DLT probability by Day 21 closest to $p^{*}=0.25$. A maximum of N=30 participants will be enrolled. Participants will be enrolled in cohorts of size C=1 and a schedule cannot be assigned to a participant unless at least $N_{1}=1$ participants have already been assigned to all lower schedules and at least $N_{2}=1$ participants have been fully followed on all lower schedules. Participant enrollment is assumed to follow a Poisson process with mean 21 days, that is, on average, each participant is enrolled after the previous participant has completed their follow‐up.

Based upon $D_{11}=0.03$, and $OR_{w}=OR_{b}=1.5$, the skeleton values assigned to each dose are shown in the first six rows of Table 1. Based upon these skeleton values, we assign β a prior normal distribution with mean $\mu_{1}=0.91$ and SD $\sigma =\sqrt{\mu_{1}}=0.95$. Assuming a value k=1.6 defined in Section 2.5, we assign $\theta_{2}$ a prior exponential distribution with mean $\mu_{2}=0.23$ and $\theta_{3}$ a prior exponential distribution with mean $\mu_{3}=0.29$.

**FIGURE 1:**
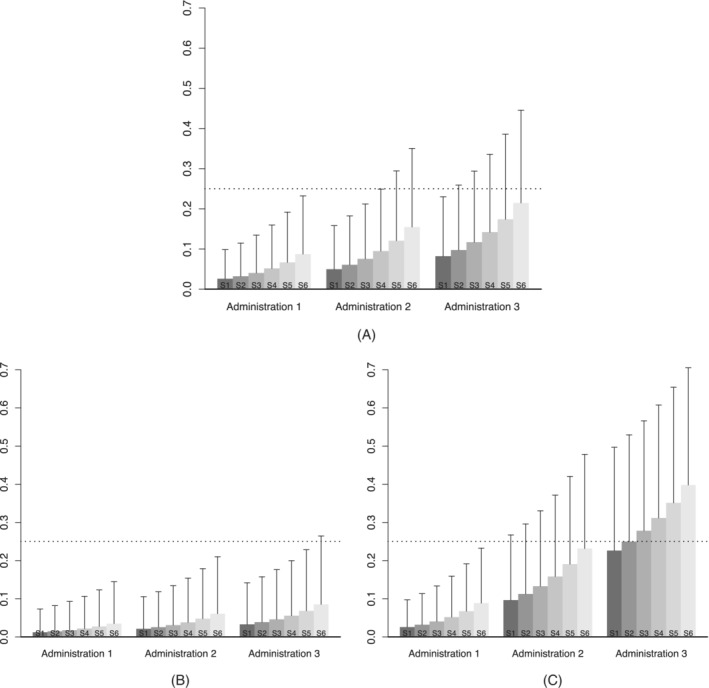
Visual representations of posterior DLT probability distributions resulting from three sets of hyperparameter values examined in Section 3. The height of each bar represents the posterior mean, and the length of each vertical line above the bar indicates the posterior SD. The horizontal dotted line represents targeted DLT probability of 0.25. S1 = schedule 1, S2 = schedule 2, …, S6 = schedule 6

Figure 1A graphically presents the prior mean and SD for the DLT probabilities for each administration of each schedule that result from the hyperparameter and skeleton values just described. In this figure, we see that all three administrations of Schedules 1 and 2 are *a priori* likely to be safe, while Schedules 4‐6 are less likely to be so. Specifically, although each has a mean prior probability of DLT at Day 21 that is below the target of 0.25, all of the latter three schedules have sufficient variability so that much of their prior distributions exceed the target.

To demonstrate how the chosen hyperparameter values impact the resulting prior distributions for DLT probabilities, Figure 1B displays information analogous to Figure 1A, but when the prior mean for β is 2.5 times larger. We now see stronger prior belief that all six schedules are safe, which might be implausible and lead to poor operating characteristics in a setting where Schedules 1 or 2 are the only acceptable schedules. Conversely, Figure 1C displays the resulting prior distributions when the prior means of $\theta_{2}$ and $\theta_{3}$ are each 2.5 larger. Now we see that there is greater prior uncertainty placed on all six schedules and we felt this prior would provide insufficient support to an algorithm incorporating data from only 30 participants. It is through these visual examinations that one can develop a suitable set of hyperparameter values before running any simulations.

To assess the operating characteristics of our design, we have simulated 1000 hypothetical trials for each of six scenarios, in which schedule j=1,2,…6 has a cumulative DLT rate after the third administration closest to $p^{*}$ in scenario j. We also include a seventh scenario in which all schedules have Day 21 DLT probabilities above the target. The true DLT rates for each scenario are shown in Table 1. These DLT probabilities are not based on any specific model; values were selected so that neighboring schedules had DLT probabilities after the third administration spaced by approximately 10 percentage points, a difference that is biologically plausible, but is also large enough to allow for discriminating between the optimal schedule and its neighbors.

### 3.3 Details for comparator approaches

We will compare the operating characteristics of our design to two other designs, both of which model a hazard function for the time‐to‐DLT and implicitly generate a cumulative probability of DLT by Day 21. Note that we have modified both methods from what was originally published in order to (i) accommodate use of the skeleton values used with our proposed model, and (ii) allow for dose levels that vary within each schedule. For the first comparator, we generalize the work of Braun et al,^19^, ^20^ assuming each administration of the drug has a hazard function at time u equal to

(4) $$h(u\;|\;\theta_{1},\theta_{2},\theta_{3})=\left\{\begin{array}{lcl} \theta_{2}\;\frac{u}{\theta_{1}} & & 0\leq u\leq \theta_{1}\\ \theta_{2}\;\frac{\theta_{3}-u}{\theta_{3}-\theta_{1}} & & \theta_{1}<u\leq \theta_{3}\\ 0 & & u>\theta_{3}\;\;\text{or}\;\;u<0, \end{array}\right.$$

which increases linearly to a value $\theta_{2}$ at time $\theta_{1}$ and then decreases linearly to zero at time $\theta_{3}$.

If a participant is assigned to a schedule of doses $d_{1},d_{2},\ldots ,d_{K}$ and dose $d_{k},k=1,2,\ldots K$ is administered at time $t_{k}$, then the total hazard of DLT at future time u is $H(u\;|\;\theta_{1},\theta_{2},\theta_{3})=\sum_{k=1}^{K}d_{k}h(u-t_{k}\;|\;\theta_{1},\theta_{2},\theta_{3}),$ which leads to a cumulative hazard function equal to $\Lambda (u\;|\;\theta_{1},\theta_{2},\theta_{3})=\int_{0}^{u}\;H(t\;|\;\theta_{1},\theta_{2},\theta_{3})\;dt$ and cumulative probability of DLT $F(u\;|\;\theta_{1},\theta_{2},\theta_{3})=1-\exp\{-\Lambda (u\;|\;\theta_{1},\theta_{2},\theta_{3})\}.$. The likelihood contribution at u is $f{\left(u\;|\;\theta_{1},\theta_{2},\theta_{3}\right)}^{Y(u)}{\left\{1-F(u\;|\;\theta_{1},\theta_{2},\theta_{3})\right\}}^{1-Y(u)},$ where Y(u)=1 if a DLT has occurred at u and is zero otherwise, and $f(u\;|\;\theta_{1},\theta_{2},\theta_{3})=dF(u\;|\;\theta_{1},\theta_{2},\theta_{3})/du.$

In our simulations, we assume a fixed value of $\theta_{3}=10$, while $\theta_{1}/10$ has a prior Beta distribution with parameters a=5.8 and b=3.9, and $\theta_{2}$ has an exponential distribution with mean 0.09. These values were selected so that the expected prior cumulative DLT probability at Day 21 for each schedule was similar to that used with the other methods.

For the second comparator, we generalized the work of Gunhan et al,^22^ replacing Equation (4) above with the difference of two exponential decay functions

$$h(u\;|\;\theta_{1},\theta_{2},\theta_{3})=\frac{\theta_{1}\theta_{2}}{\theta_{2}-\theta_{3}}[exp\{-\theta_{3}(u-t_{k})\}-exp\{-\theta_{2}(u-t_{k}\}].$$

All remaining equations above for the total and cumulative hazards and the cumulative probability of DLT are unchanged. In the simulations, we have assumed fixed values for both $\theta_{2}$ and $\theta_{3}$, such that $\theta_{2}=0.14$ and $\theta_{3}=0.35$. With regard to $\theta_{1}$, we assume $\log(\theta_{1})$ has a normal distribution with mean −2 and SD 1, again so that the expected prior cumulative DLT probability of each schedule was similar to that of the other methods.

For both comparators, we use the same stopping rule and limits on escalation as used with our model, so that variations in the operating characteristics presented next are due primarily to the model that is used. All simulations were performed in R, version 4.0.4. Due to a vast savings in computation time, all posterior moments were computed through integral approximation methods, rather than through Markov Chain Monte Carlo methods. All code for our method, as well as the two comparator methods, is available on GitHub at https://github.com/tombraun1216/CRM‐with‐Step‐Up‐Dosing.git.

### 3.4 Operating characteristics

**FIGURE 2:**
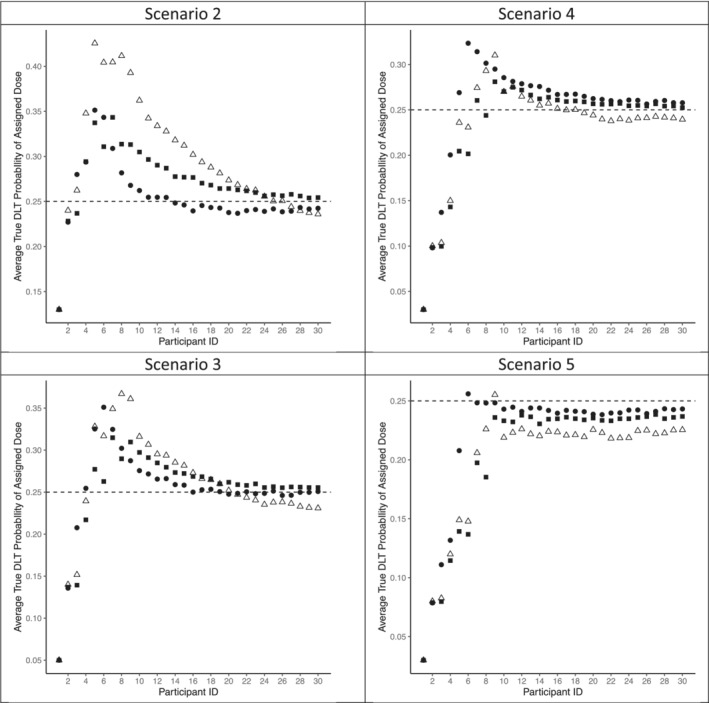
Schedule assignment patterns in Scenarios 2‐5 of simulations described in Section 3. •, proposed method using extension of CRM; ■, PK hazard method of Reference 22; ▵, triangular hazard method of References 19 and 20. Horizontal dashed line represents targeted DLT probability of .25

The performance of our design is summarized by three metrics: (i) the proportion of simulations in which each schedule is selected as the MTS at the end of the study; (ii) the average proportion of participants assigned to each schedule during the study, and (iii) the average Day 21 DLT probability of the schedule assigned to each participant. Information regarding metrics (i) and (ii) is shown in Table 2, while information regarding metric (iii) is shown in Figure 2.

**TABLE 2:** Operating characteristics resulting from simulations described in Section 3.2

		Early term			Schedule 1			Schedule 2			Schedule 3			Schedule 4			Schedule 5			Schedule 6	
Scen	Method	Sel	Assn		Sel	Assn		Sel	Assn		Sel	Assn		Sel	Assn		Sel	Assn		Sel	Assn
1	CRM	0.08	n/a		**0.68**	**0.66**		0.20	0.17		0.05	0.09		0.00	0.03		0.00	0.01		0.00	0.00
	GWF	0.07	n/a		**0.60**	**0.51**		0.28	0.28		0.05	0.13		0.00	0.03		0.00	0.00		0.00	0.00
	TRI	0.06	n/a		**0.72**	**0.39**		0.16	0.27		0.06	0.25		0.00	0.06		0.00	0.01		0.00	0.00
2	CRM	0.01	n/a		0.32	0.38		**0.40**	**0.29**		0.23	0.21		0.04	0.09		0.00	0.03		0.00	0.01
	GWF	0.00	n/a		0.21	0.23		**0.49**	**0.40**		0.26	0.26		0.04	0.10		0.00	0.02		0.00	0.00
	TRI	0.00	n/a		0.42	0.19		**0.28**	**0.27**		0.27	0.38		0.03	0.13		0.00	0.02		0.00	0.00
3	CRM	0.00	n/a		0.02	0.12		0.25	0.22		**0.41**	**0.31**		0.27	0.23		0.04	0.09		0.00	0.03
	GWF	0.00	n/a		0.00	0.06		0.23	0.24		**0.45**	**0.35**		0.28	0.26		0.04	0.08		0.00	0.01
	TRI	0.00	n/a		0.12	0.08		0.15	0.16		**0.46**	**0.39**		0.24	0.29		0.03	0.07		0.00	0.01
4	CRM	0.00	n/a		0.00	0.06		0.03	0.09		0.21	0.19		**0.42**	**0.29**		0.30	0.24		0.05	0.13
	GWF	0.00	n/a		0.00	0.04		0.02	0.11		0.25	0.22		**0.44**	**0.34**		0.25	0.21		0.05	0.07
	TRI	0.00	n/a		0.04	0.04		0.02	0.10		0.23	0.22		**0.42**	**0.38**		0.27	0.20		0.02	0.06
5	CRM	0.00	n/a		0.00	0.05		0.01	0.07		0.06	0.11		0.29	0.22		**0.41**	**0.28**		0.24	0.26
	GWF	0.00	n/a		0.00	0.04		0.01	0.09		0.07	0.13		0.31	0.27		**0.40**	**0.28**		0.22	0.18
	TRI	0.00	n/a		0.02	0.04		0.01	0.09		0.07	0.12		0.32	0.31		**0.43**	**0.28**		0.16	0.16
6	CRM	0.00	n/a		0.00	0.05		0.00	0.05		0.02	0.08		0.12	0.14		0.34	0.24		**0.52**	**0.44**
	GWF	0.00	n/a		0.00	0.04		0.00	0.08		0.02	0.09		0.14	0.19		0.33	0.26		**0.51**	**0.34**
	TRI	0.00	n/a		0.01	0.04		0.00	0.08		0.02	0.09		0.16	0.22		0.39	0.27		**0.42**	**0.31**
7	CRM	0.75	n/a		0.24	0.48		0.00	0.05		0.00	0.02		0.00	0.01		0.00	0.00		0.00	0.00
	GWF	0.71	n/a		0.28	0.47		0.02	0.11		0.00	0.03		0.00	0.01		0.00	0.00		0.00	0.00
	TRI	0.57	n/a		0.41	0.38		0.02	0.22		0.01	0.16		0.00	0.02		0.00	0.00		0.00	0.00

*Note*: CRM, proposed extension of CRM; GWF, PK hazard method of Reference 22; TRI, triangular hazard method of References 19 and 20; Sel, proportion of simulations in which schedule was selected as best at end of study; Assn, average proportion of participants assigned to schedule during study; early term, no schedule selected due to early termination of study. Boldface text corresponds to operating characteristics corresponding to schedule with true DLT closest to target DLT probability of .25.

To start, we focus on the boldfaced values in Table 2, which indicate that final selection or assignment during the study is done at the schedule with Day 21 DLT probability closest to the target. With regard to the decision made at the end of the study, we see that all three methods have fairly similar performance; averaging across all scenarios, we have correct selection proportions of 0.47, 0.48, and 0.46 for our proposed method, the method of Gunhan et al, and the method of Braun et al, respectively. Furthermore, we extended the work of O'Quigley et al,^29^ and generated, for each of the first six scenarios, a non‐parametric optimal upper bound for probability of correct schedule selection at study end. Based upon 10 000 simulations, the realized upper bounds were 0.69, 0.57, 0.52, 0.49, 0.54, and 0.58 for scenarios 1‐6, respectively, with an average of 0.57, which supports that all three methods have solid, but not overly optimistic, performance with 30 participants.

With regard to assignments made during the trial, 2 presents fairly comparable performance among the methods, although with greater variation than what was seen for the probability of correct selection at study end. Averaging across all six scenarios, we have average proportions of assignment equal to 0.38, 0.37, and 0.34 for our proposed method, the method of Gunhan et al, and the method of Braun et al, respectively. The greatest discrepancy among the methods appears in Scenario 2, in which both our proposed design and the triangular hazard design both assign fewer participants to schedule 2 than does the PK‐based hazard design. This issue is also demonstrated in the upper left‐hand panel in Figure 2, in which we see that the proposed design tends to assign Schedule 1 more often than Schedule 2, while the triangular hazard design tends to assign Schedule 3 more than Schedule 2.

To examine this phenomenon in more detail, we examined all six scenarios and tabulated, for each of the three designs, the difference in schedule assignments between a current participant who experiences a DLT and the next participant, that is, determining if the next assignment was at a lower schedule, the same schedule, or a higher schedule. What we found was that across the six scenarios, the probability of de‐escalating by two or more schedule levels ranged from 0.05 to 0.13 for our proposed design, 0.01 to 0.03 for the PK hazard design, and 0.01 to 0.04 for the triangular hazard design. Conversely, the probability of escalating one schedule level ranged from 0.01 to 0.02 for our proposed design, 0.01 to 0.03 for the PK hazard design, and 0.07 to 0.11 for the triangular hazard design.

This differential is also supported by the results in Table 2 for scenario 7, in which all six schedules are overly toxic, so that dose assignments should be restricted to the lowest schedules, and the study should terminate before accruing all 30 participants. We see that the proposed method and PK‐based hazard method both terminate accrual more often than the triangular hazard approach, which also assigns schedule 3 far more often than the others. Furthermore, early termination occurred after an accrual of 13, 14, and 19 participants, on average, for our method, the PK hazard design, and the triangular hazard design, respectively.

In Figure 2, we have presented the dose assignment patterns for Scenarios 2 to 5 and have omitted Scenarios 1 and 6 because they present redundant information from the other scenarios. We see across all four scenarios that all three methods have assignments that tend to coverage toward the schedule with a DLT probability closest to the target of .25. As expected, all three methods tend to escalate to schedules with DLT probabilities higher than .25, and then respond to observed DLTs and de‐escalate in efforts to observe fewer DLTs. The greatest divergence among the three methods is seen for Scenario 2 and supports our earlier discussion of the results presented in Table 2, namely that our proposed method tends to be least aggressive with schedule assignments and the triangular hazard method tends to be most aggressive.

### 3.5 Sensitivity analyses

Using the settings from Scenarios 1 to 6 presented in Table 1, we also examined how the operating characteristics of our proposed methods varied with (i) a different prior distribution for $\theta_{2}$ and $\theta_{3}$, (ii) a different skeleton, which implicitly impacts the prior for β, (iii) a larger sample size of 45 participants, and (iv) a shorter average inter‐arrival for participants of seven days. The results for aspects (i) and (ii) can be found in Table 3, while the results for aspects (iii) and (iv) can be found in Table S1 in the Appendix.

**TABLE 3:** Simulation results for assessing sensitivity to prior distributions and skeleton

		Sched 1			Sched 2			Sched 3			Sched 4			Sched 5			Sched 6	
Sensitivity feature	Scen	Sel	Assn		Sel	Assn		Sel	Assn		Sel	Assn		Sel	Assn		Sel	Assn
Prior for $\theta_{2}$ and $\theta_{3}$	1	**0.69**	**0.69**		0.19	0.18		0.05	0.07		0.00	0.02		0.00	0.00		0.00	0.00
	2	0.33	0.42		**0.40**	**0.28**		0.22	0.19		0.04	0.08		0.00	0.02		0.00	0.00
	3	0.02	0.15		0.25	0.23		**0.42**	**0.31**		0.26	0.21		0.05	0.08		0.00	0.02
	4	0.00	0.08		0.04	0.11		0.23	0.22		**0.43**	**0.29**		0.26	0.21		0.04	0.10
	5	0.00	0.07		0.01	0.08		0.06	0.13		0.31	0.24		**0.41**	**0.27**		0.20	0.23
	6	0.00	0.05		0.00	0.06		0.03	0.10		0.14	0.16		0.35	0.24		**0.48**	**0.39**
Skeleton dose values	1	**0.69**	**0.61**		0.21	0.22		0.03	0.09		0.00	0.03		0.00	0.01		0.00	0.00
	2	0.28	0.31		**0.47**	**0.35**		0.22	0.23		0.03	0.08		0.00	0.02		0.00	0.00
	3	0.01	0.11		0.23	0.21		**0.46**	**0.34**		0.27	0.25		0.03	0.07		0.00	0.01
	4	0.00	0.05		0.02	0.09		0.22	0.20		**0.47**	**0.34**		0.27	0.24		0.03	0.08
	5	0.00	0.04		0.01	0.07		0.07	0.13		0.36	0.27		**0.44**	**0.31**		0.13	0.17
	6	0.00	0.04		0.00	0.05		0.03	0.10		0.18	0.19		0.42	0.31		**0.36**	**0.32**

*Note*: Sel, proportion of simulations in which schedule was selected as best at end of study; Assn, average proportion of participants assigned to schedule during study; boldface text corresponds to operating characteristics corresponding to schedule with true DLT closest to target DLT probability of .25.

Assuming an increased value k=3.2 (as described in Section 2.5), we assigned $\theta_{2}$ a prior exponential distribution with mean $\mu_{2}=0.38$ and $\theta_{3}$ a prior exponential distribution with mean $\mu_{3}=0.61$. From the first set of six rows in Table 3, we also see no substantial change to the operating characteristics for our design, although with slightly lower performance in scenario 6. The latter set of six rows in Table 3 correspond to operating characteristics when we changed the skeleton by using values $D_{11}=0.01$, and $OR_{w}=OR_{b}=2.0$ (as described in Section 2.4), which led to skeleton values that were shifted closer to zero. As with the prior distributions for $\theta_{2}$ and $\theta_{3}$, we see little change in operating characteristics for scenarios 1 to 5, although the performance is decreased in scenario 6, which emphasizes that the skeleton values do impact the prior mean for $\beta_{1}$, which is the likely cause of this result in scenario 6.

In Supplementary Table S1, the first six rows present the operating characteristics when the sample size is increased to 45 participants. Not surprisingly, we see that the probability of correctly selecting the best schedule increases approximately 10 points, with a slightly lower increase in the average proportion of participants assigned to the best schedule. With regard to the average inter‐arrival time of patients, when participants arrived an average of every 7 days, rather than every 21 days, the last six rows of Supplementary Table S1 demonstrate no material change in the operating characteristics relative to those in Table 2.

## 4 DISCUSSION

In recent Phase I dose‐escalation trials assessing the tolerability of new investigational drugs in cancer immunotherapy, high rates of DLT after first dose have been reported. To address this issue, adjusted dosing schemes have been proposed that consist of planned stepwise dose‐escalation for each participant at the start of treatment. These dosing schemes, known as SUD or dose fractionation, pose new challenges for the design of such a Phase I dose‐escalation trial.

Certainly, identifying the MTD in a Phase I dose‐escalation trial is always challenging because the process relies on sparse data. Each patient contributes a single data point, either a 0 if no DLT is observed during the DLT assessment period, or a 1 otherwise. With SUD schemes, the challenge of identifying the MTD is even larger, as a combination of doses have to be identified, each of which induces a probability of toxicity close to the target toxicity level.

To this end, we have proposed an approach for assessing the cumulative probability of DLT for a series of administrations given to participants in a SUD design, whereby participants are first given administrations of a lower dose in hopes of leading to less likelihood of DLT when the final desired dose of the agent is administered. Our design is a simple extension of the CRM, as compared to other approaches founded in time‐to‐event methods, and our design has operating characteristics comparable to those methods. Our method resembles a piecewise hazard model and is suitable for designs in which participants are treated with a succession of non‐decreasing doses. One limitation of our method, is that each administration is assumed to occur within a specific timeframe, so our current model is unable to accommodate treatment delays and resulting DLT information, unless additional assumptions or modifications are made.

The number of parameters in our model also grows with the number of administrations, although we could include approaches to smooth among the parameters, correlate them through their prior distributions, or even assume that the change in DLT probability is the same for each additional administration. Nonetheless, our method does not make an assumption of additivity of hazards among the administrations, as is done with the approaches we used as comparators. And, like all designs using Bayesian methods, any data from a previous study of a related compound, including information on PK and PD patterns, should be considered to assist with the selection of prior distributions and all parameter and dose values.

Our proposed CRM extension requires specifying a skeleton, which is a set of plausible values ascribed to each dose and is often related to the *a priori* probability of DLT for single administration. Typically, the skeleton is chosen to cover a wide range of possible dose‐toxicity profiles, and this choice can be difficult given the lack of prior knowledge on dose‐toxicity at the study start. To help in this undertaking, we provide a systematic and intuitive approach for choosing the values used in the skeleton. By simplifying the selection of the initial guesses of the probabilities of DLT in practice, we hope to enhance the use of our CRM‐based approach.

Our method is also extremely flexible and allows for nearly any modification desired for a specific trial. For example, although our simulations assigned a dose to each participant individually, such assignments can be done to a collective cohort of participants, such as in groups of three participants, as a safeguard against early escalation to doses later seen to be toxic. Our method also allows for any dose‐toxicity model used in the CRM, such as a standard logistic model with known intercept.

Nonetheless, before implementing our or any proposed design, clinical and safety experts should be consulted to evaluate the risk of planning to increase a participant's dose while on study. Additionally, a thoughtful and careful safety monitoring plan should be made and possibly include restrictions on how many participants can be exposed to a dose level, whether a participant is exposed to that dose initially as part of a new study cohort or is exposed during their planned dose escalation. Furthermore, our design is founded on a strong *a priori* belief that SUD is necessary. Although our design does allow for the estimation of DLT probabilities for single administrations of varying doses, as well as the ability to compare the DLT probability of a dose given immediately vs gradually via SUD, investigators must weight scientific interest in this latter comparison with the safety, costs, and time resources that might arise with repeated administrations.

## Supporting information


**Table S1**: Simulation results for assessing sensitivity to sample size and inter‐arrival times. Sel = proportion of simulations in which schedule was selected as best at end of study; Assn = average proportion of participants assigned to schedule during study; boldface text corresponds to operating characteristics corresponding to schedule with true DLT closest to target DLT probability of 0.25.

## Data Availability

Data sharing is not applicable to this article as no new data were created or analyzed.

## 1

### A.1 DERIVING CONDITIONAL PROBABILITIES

To promote readability, all derivations below omit conditioning upon doses.

For the second administration, we have

$$\begin{array}{ll} \pi_{i2}=Pr(Y_{i2}=1) & =Pr(Y_{i2}=1\;|\;Y_{i1}=0)\times Pr(Y_{i1}=0)\;\\ & \;+Pr(Y_{i2}=1\;|\;Y_{i1}=1)\times Pr(Y_{i1}=1)\;\\ & =Pr(Y_{i2}=1\;|\;Y_{i1}=0)\times (1-\pi_{i1})+1\times \pi_{i1}.\; \end{array}$$

Solving for $Pr(Y_{i2}=1\;|\;Y_{i1}=0)$, we have

$$\begin{array}{ll} Pr(Y_{i2}=1\;|\;Y_{i1}=0)=\frac{\pi_{i2}-\pi_{i1}}{1-\pi_{i1}}. & \end{array}$$

This conditional probability also leads to the joint probability

$$\begin{array}{ll} Pr(Y_{i2}=1,Y_{i1}=0) & =Pr(Y_{i2}=1\;|\;Y_{i1}=0)\times [1-Pr(Y_{i1}=1)]\;\\ & =\pi_{i2}-\pi_{i1},\; \end{array}$$

which will be used in the following derivation.

For the third administration, we have

$$\begin{array}{ll} Pr(Y_{i3}=1) & =Pr(Y_{i3}=1\;|\;Y_{i2}=1,Y_{i1}=0)\times Pr(Y_{i2}=1,Y_{i1}=0)\;\\ & \;+Pr(Y_{i3}=1\;|\;Y_{i2}=0,Y_{i1}=0)\times Pr(Y_{i2}=0,Y_{i1}=0)\;\\ & \;+Pr(Y_{i3}=1\;|\;Y_{i2}=1,Y_{i1}=1)\times Pr(Y_{i2}=1,Y_{i1}=1)\;\\ & \;+Pr(Y_{i3}=1\;|\;Y_{i2}=0,Y_{i1}=1)\times Pr(Y_{i2}=1,Y_{i1}=1)\;\\ & \;\\ & =Pr(Y_{i2}=1,Y_{i1}=0)\;\\ & \;+Pr(Y_{i3}=1\;|\;Y_{i2}=0,Y_{i1}=0)\times Pr(Y_{i2}=0,Y_{i1}=0)\;\\ & \;+Pr(Y_{i2}=1\;|\;Y_{i1}=1)\times Pr(Y_{i1}=1)\;\\ & \;\\ & =Pr(Y_{i2}=1,Y_{i1}=0)\;\\ & \;+Pr(Y_{i3}=1\;|\;Y_{i2}=0,Y_{i1}=0)\times Pr(Y_{i2}=0,Y_{i1}=0)\;\\ & \;+Pr(Y_{i1}=1),\; \end{array}$$

because $Pr(Y_{i3}=1\;|\;Y_{i2}=1,Y_{i1}=0)$, $Pr(Y_{i3}=1\;|\;Y_{i2}=1,Y_{i1}=1)$, and $Pr(Y_{i2}=1\;|\;Y_{i1}=1)$ are each exactly equal to 1, and $Pr(Y_{i3}=1\;|\;Y_{i2}=0,Y_{i1}=1)$ is exactly equal to 0.

Solving for $Pr(Y_{i3}=1\;|\;Y_{i2}=0,Y_{i1}=0)$, we have

$$\begin{array}{ll} Pr(Y_{i3}=1\;|\;Y_{i2}=0,\;Y_{i1}=0) & =\frac{Pr(Y_{i3}=1)-Pr(Y_{i2}=1,\;Y_{i1}=0)-Pr(Y_{i1}=1)}{Pr(Y_{i2}=0,\;Y_{i1}=0)}\;\\ & \;\\ & =\frac{\pi_{i3}-(\pi_{i2}-\pi_{1i})-\pi_{1i}}{Pr(Y_{i2}=0\;|\;Y_{i1}=0)\times Pr(Y_{i1}=0)}\;\\ & \;\\ & =\frac{\pi_{i3}-\pi_{i2}}{\left[1-Pr(Y_{i2}=1\;|\;Y_{i1}=0)\right]\times \left[1-Pr(Y_{i1}=1)\right]}\;\\ & \;\\ & =\frac{\pi_{i3}-\pi_{i2}}{\left[(1-\pi_{i2})\;/\;(1-\pi_{i1})]\times (1-\pi_{i1}\right)}\;\\ & \;\\ & =\frac{\pi_{i3}-\pi_{i2}}{1-\pi_{i2}}.\; \end{array}$$

### A.2 Deriving prior mean for model parameter β

We start with $f(\beta )=\pi_{j1}=d_{j1}^{exp(\beta )}$. Given first and second derivatives

$$\begin{array}{ll} f^{\prime }(\beta )=\frac{df}{d\beta } & =\log(d_{j1})exp(\beta )f(\beta )\;\\ f^{\prime \prime }(\beta )=\frac{d^{2}f}{d\beta^{2}} & =\log(d_{j1})exp(\beta )[f^{{}^{\prime }}(\beta )+f(\beta )]\;\\ & =\log(d_{j1})exp(\beta )f(\beta )[\log(d_{j1})exp(\beta )+1]\; \end{array}$$

we use a second order Taylor expansion of f(β) around the prior mean $\mu_{1}$ to derive

$$\begin{array}{ll} E[f(\beta )] & =f(\mu_{1})\;\\ & \;+0.5\sigma^{2}\log(d_{j1})exp(\mu_{1})f(\mu_{1})[\log(d_{j1})exp(\mu_{1})+1].\; \end{array}$$

We seek to make the second‐order term equal to zero, which occurs when $\log(d_{j1})exp(\mu_{1})=-1$. Solving for $\mu_{1}$, we find the suggested prior mean $\mu_{1}=\log[-\log(d_{j1})]$. To collapse over all schedules, we compute the arithmetic average, so that we have

$$\mu_{1}=\frac{1}{6}\overset{6}{\underset{j=1}{\sum }}\log\left[-\log(d_{j1})\right].$$
