# Supplementary material for: Extending the Continual Reassessment Method to accommodate step‐up dosing in Phase I trials
Source: Stat Med. 2022 Jun 5;41(20):3975–90. doi: 10.1002/sim.9487 (PMC9546169; doi:10.1002/sim.9487)
Supplement: Supplementary file 1 — Table S1: Simulation results for assessing sensitivity to sample size and inter‐arrival times. Sel = proportion of simulations in which schedule was selected as best at end of study; Assn = average proportion of participants assigned to schedule during study; boldface text corresponds to operating characteristics corresponding to schedule with true DLT closest to target DLT probability of 0.25. [file SIM-41-3975-s001.pdf]

Table S1 (Supplementary): Simulation results for assessing sensitivity to sample size and inter-arrival times. Sel = proportion of simulations in which schedule was selected as best at end of study; Assn = average proportion of participants assigned to schedule during study; boldface text corresponds to operating characteristics corresponding to schedule with true DLT closest to target DLT probability of 0.25.

		Sched 1		Sched 2		Sched 3		Sched 4		Sched 5		Sched 6	
Sensitivity Feature	Scen	Sel	Assn	Sel	Assn	Sel	Assn	Sel	Assn	Sel	Assn	Sel	Assn
Sample Size	1	**0.71**	**0.67**	0.18	0.18	0.02	0.07	0.00	0.02	0.00	0.01	0.00	0.00
	2	0.24	0.34	**0.52**	**0.35**	0.22	0.22	0.03	0.06	0.00	0.02	0.00	0.00
	3	0.01	0.10	0.20	0.20	**0.52**	**0.36**	0.25	0.24	0.02	0.07	0.00	0.02
	4	0.00	0.04	0.01	0.07	0.19	0.20	**0.49**	**0.34**	0.29	0.25	0.02	0.10
	5	0.00	0.04	0.00	0.06	0.03	0.10	0.27	0.24	**0.50**	**0.33**	0.20	0.22
	6	0.00	0.03	0.00	0.04	0.01	0.08	0.08	0.13	0.37	0.27	**0.55**	**0.46**
Inter-arrival times	1	**0.66**	**0.68**	0.20	0.17	0.06	0.07	0.00	0.02	0.00	0.00	0.00	0.00
	2	0.29	0.39	**0.39**	**0.30**	0.26	0.20	0.05	0.08	0.00	0.02	0.00	0.00
	3	0.02	0.14	0.19	0.23	**0.43**	**0.29**	0.30	0.22	0.05	0.09	0.00	0.02
	4	0.00	0.09	0.02	0.13	0.19	0.18	**0.39**	**0.25**	0.33	0.23	0.07	0.12
	5	0.00	0.09	0.00	0.12	0.05	0.13	0.27	0.20	**0.41**	**0.25**	0.26	0.22
	6	0.00	0.08	0.00	0.11	0.02	0.10	0.12	0.15	0.32	0.21	**0.54**	**0.35**
